# Comment on Long‐Term Outcomes of Catheter Ablation in Ventricular Tachycardia Electrical Storm

**DOI:** 10.1002/clc.70265

**Published:** 2026-01-24

**Authors:** Sohana Memon, Gaaitri Lohano

**Affiliations:** ^1^ Liaquat University of Medical and Health Sciences (LUMHS) Jamshoro Pakistan

Dear Editor,

The research by Çöteli et al. [1], titled *“Long‐Term Outcomes of Catheter Ablation in Ventricular Tachycardia Electrical Storm: A Retrospective Cohort Study,”* caught our interest. The authors deserve praise for their methodical evaluation of a high‐risk patient population presenting with electrical storm and for providing a comprehensive description of their retrospective cohort study design, patient selection criteria, and procedural methods. Their detailed documentation of VT induction strategies, ICD therapy monitoring, catheter ablation techniques, and follow‐up procedures offers valuable insight into real‐world management. These methodological strengths reinforce the reliability of the reported findings regarding procedural success, VT recurrence, ICD interventions, and survival outcomes. Nevertheless, certain limitations related to risk stratification and outcome assessment may influence the interpretation and generalizability of the results.

First, in high‐risk cardiac populations, procedural tolerance, mortality, and post‐ablation outcomes are influenced not only by left ventricular ejection fraction and comorbidities but also by overall physiological reserve. Patients with similar LVEF profiles may differ substantially in frailty status, which has been shown to be an independent predictor of cardiovascular mortality. The absence of frailty assessment in the current study limits granular risk stratification and may complicate interpretation of long‐term mortality and morbidity outcomes. Although frailty evaluation can be challenging in retrospective cohorts, incorporating validated frailty measures in future studies could enhance prognostic accuracy and clinical applicability [2].

Second, an important methodological consideration is the lack of a standardized ventricular tachycardia induction protocol during catheter ablation. While programmed ventricular stimulation and burst pacing were employed, details regarding pacing sites, number of extrastimuli, and stimulation parameters were not uniformly defined. Such variability may influence VT detection and the prognostic interpretation of post‐ablation non‐inducibility, even if it does not undermine procedural efficacy itself. Prior high‐risk VT ablation studies have demonstrated that variability in stimulation methodology can affect the predictive value of inducibility for long‐term clinical outcomes [3].

Finally, the influence of operator experience on procedural outcomes was not addressed. Given the prolonged study period and the complexity of VT ablation procedures, including combined endocardial and epicardial approaches, operator‐related variability may have affected outcomes. Recent evidence demonstrates that higher procedural volume is associated with improved safety and efficacy in VT ablation: Bansal et al. (2025) showed that high‐volume centers had significantly lower in‐hospital mortality and major complications compared with low‐volume centers, highlighting the importance of operator and institutional experience in procedural outcomes [4].

Once again, we sincerely appreciate the authors' valuable contribution to the literature and their efforts in addressing an important and challenging clinical condition. However, as researchers, we believe that there is always space for improvement and that any necessary adjustments should be made since they would improve our comprehension of the research as a whole.

## Funding

The authors received no specific funding for this work.

## Ethics Statement

Ethical approval does not apply to such articles.

## Conflicts of Interest

The authors declare no conflicts of interest.

## Data Availability

The authors have nothing to report.

## References

[1] C. Çöteli , S. Zekeriyayev , C. Sezer , H. Yorgun , and K. Aytemir , “Long‐Term Outcomes of Catheter Ablation in Ventricular Tachycardia Electrical Storm: A Retrospective Cohort Study,” Clinical Cardiology 48, no. 11 (2025): e70221, 10.1002/clc.70221. 41241775 PMC12619898

[2] Y. Zhao , Y. Wu , Z. Liu , and A. Zhu , “Association of Frailty and Pre‐Frailty With Cardiovascular Mortality: A Meta‐Analysis of 26 Cohort Studies,” Frontiers in Public Health 13 (2025): 1688014, 10.3389/fpubh.2025.16880143. 41323622 PMC12658331

[3] J. Sipko , B. Baranowski , M. Bhargava , et al., “Acute Post‐Procedural Inducibility Is a Poor Predictor of Clinical Outcomes in High‐Risk Patients (PAINESD > 17) Undergoing Scar‐Related Ventricular Tachycardia Ablation,” Europace: European Pacing, Arrhythmias, and Cardiac Electrophysiology: Journal of the Working Groups on Cardiac Pacing, Arrhythmias, and Cardiac Cellular Electrophysiology of the European Society of Cardiology 26, no. 7 (2024): euae185, 10.1093/europace/euae185. 39031021 PMC11259852

[4] A. Bansal , A. Nandan , J. Sroubek , et al., “Impact of Hospital VT Ablation Volume on Postprocedural Complications: Argument for Selective Referral to High‐Volume Centers,” JACC: Clinical Electrophysiology 11, no. 7 (2025): 1453–1461, 10.1016/j.jacep.2025.02.041. 40338775

